# Contralateral parenchymal enhancement on dynamic contrast-enhanced MRI reproduces as a biomarker of survival in ER-positive/HER2-negative breast cancer patients

**DOI:** 10.1007/s00330-018-5470-7

**Published:** 2018-05-07

**Authors:** Bas H. M. van der Velden, Elizabeth J. Sutton, Luca A. Carbonaro, Ruud M. Pijnappel, Elizabeth A. Morris, Kenneth G. A. Gilhuijs

**Affiliations:** 1 Image Sciences Institute, University Medical Center Utrecht, Heidelberglaan 100, 3584 CX Utrecht, The Netherlands; 2 Department of Radiology, Memorial Sloan Kettering Cancer Center, 1275 York Avenue, New York, NY 10065 USA; 3 Department of Radiology, IRCCS Policlinico San Donato, Piazza Edmondo Malan 2, 20097 San Donato Milanese, Milan Italy; 4 Department of Radiology, University Medical Center Utrecht, Heidelberglaan 100, Utrecht, The Netherlands

**Keywords:** Unilateral breast neoplasms, Magnetic resonance imaging, Parenchymal tissue, Survival analysis, Image processing, computer-assisted

## Abstract

**Objectives:**

To assess whether contralateral parenchymal enhancement reproduces as an independent biomarker for patient survival in an independent patient cohort from a different cancer institution.

**Methods:**

This is a HIPAA-compliant IRB approved retrospective study. Patients with ER-positive/HER2-negative operable invasive ductal carcinoma and preoperative dynamic contrast-enhanced MRI were consecutively included between 2005 and 2009. The parenchyma of the breast contralateral to known cancer was segmented automatically on MRI and contralateral parenchymal enhancement (CPE) was calculated. CPE was split into tertiles and tested for association with invasive disease-free survival (IDFS) and overall survival (OS). Propensity score analysis with inverse probability weighting (IPW) was used to adjust CPE for patient and tumour characteristics as well as systemic therapy.

**Results:**

Three hundred and two patients were included. The median age at diagnosis was 48 years (interquartile range, 42-57). Median follow-up was 88 months (interquartile range, 76-102); 15/302 (5%) patients died and 37/302 (13%) had a recurrence or died. In context of multivariable analysis, IPW-adjusted CPE was associated with IDFS [hazard ratio (HR) = 0.27, 95% confidence interval (CI) = 0.05-0.68, *p* = 0.004] and OS (HR = 0.22, 95% CI = 0.00-0.83, *p* = 0.032).

**Conclusions:**

Contralateral parenchymal enhancement on pre-treatment dynamic contrast-enhanced MRI as an independent biomarker of survival in patients with ER-positive/HER2-negative breast cancer has been upheld in this study. These findings are a promising next step towards a practical and inexpensive test for risk stratification of ER-positive/HER2-negative breast cancer.

**Key points:**

*• High parenchymal-enhancement in the disease-free contralateral breast reproduces as biomarker for survival.*

*• This is in patients with ER-positive/HER2-negative breast cancer from an independent cancer centre.*

*• This is independent of patient and pathology parameters and systemic therapy.*

## Introduction

Breast cancer is a heterogeneous disease with inter- and intra-tumour heterogeneity. Different molecular subtypes have been defined that are associated with disease-free and overall survival (OS). Clinically, immunohistochemical surrogates are used to guide treatment including oestrogen receptor (ER)-positive/ human epidermal growth factor receptor 2 (HER2)-negative, HER2-positive and triple-negative [1–4]. More recently, molecular assays have yielded additional tools to predict therapy outcome, helping to optimise strategies for patient-tailored treatment [5–8]. Nonetheless, using current routine predictive markers in clinical practice, treatment outcome still varies within specific subtypes. Hence, an ongoing need for accurate risk stratification exists, where risk markers are correlated with outcome after breast cancer therapy.

Current routine tests mainly focus on the tumour; meanwhile, the breast parenchyma is relatively underexplored. Genomic changes in the parenchyma may generate cell transformations leading to malignancy [9]. Hence, the parenchyma may be important for breast cancer risk prediction, but also for treatment response and assessment of outcome [10–17]. The parenchyma can be assessed using imaging and its relationship with patient outcome can be evaluated. Assuming symmetry between both breasts, one might hypothesise that the parenchyma in the disease-free breast (i.e. contralateral to the known cancer) is comparable to that in the diseased breast before tumourigenesis. Consequently, analysis of the parenchyma in the disease-free breast before treatment might gain insight into the role of the breast’s healthy parenchyma on patient outcome.

Earlier studies in patients with ER-positive/HER2-negative breast cancer showed that more pronounced contralateral parenchymal enhancement (CPE) on magnetic resonance imaging (MRI) prior to treatment was associated with improved patient survival, independent of other routine predictive markers [18, 19]. This effect was even more evident in patients receiving endocrine treatment. For proper assessment of the potential of CPE in patient stratification, this must be confirmed in an independent set of patients. Thus, the purpose of this study was to assess whether CPE, as an independent biomarker for patient survival, reproduces in an independent patient cohort from a different cancer institution.

## Materials and methods

### Data and study design

The study was Health Insurance Portability and Accountability Act-compliant and received Institutional Review Board approval. The study adheres to the REMARK guidelines [20]. In a previous study (i.e. the biomarker-discovery study), contralateral-parenchymal enhancement stratified survival of patients with ER-positive/HER2-negative breast cancer at The Netherlands Cancer Institute [18]. In the current biomarker-assessment study, we aimed to assess whether the biomarker can be confirmed in consecutive patients with ER-positive/HER2-negative breast cancer from the Memorial Sloan Kettering Cancer Center in the United States. Both studies are retrospective in nature. The following paragraphs describe the validation in more detail.

### Patient eligibility

Women treated between 2005 and 2009 were consecutively included if they (1) had unilateral ER-positive/HER2-negative invasive ductal carcinoma, (2) were eligible for breast-conserving therapy based on conventional imaging and clinical examination, and (3) received preoperative breast MRI. Patients with prior breast cancer or prior breast surgery were excluded. Additional exclusion criteria were: (1) contralateral pathology-proven benign findings and contralateral metal clips, and (2) image acquisition or registration errors, since they might influence the MRI biomarker.

### Pathology

Tumours were ER-positive if more than 1% of the cells stained positive at immunohistochemistry [21], progesterone receptor (PR)-positive if more than 1% of the cells stained positive at immunohistochemistry [21] and HER2-negative when cells scored 0 to 1+ at immunohistochemistry or scored 2+ with a negative fluorescence in situ hybridisation test. Histological grade was assessed using the Bloom and Richardson method [22]. The number of axillary lymph nodes positive for malignancy was registered. The largest tumour diameter was measured on pathology.

### Menopausal and menstrual status

Patient menopausal status was recorded as premenopausal, perimenopausal or postmenopausal. Premenopausal patients were menstruating regularly, perimenopausal less regularly with dipping oestrogen and postmenopausal not for a year. For premenopausal patients, the timing of the menstrual cycle was recorded as the number of weeks since the start of the last menstrual cycle.

### Magnetic resonance imaging

Dynamic contrast-enhanced MRI was performed using a 1.5-T unit with dedicated eight-channel breast-coil (Signa; General Electric Medical Systems, Waukesha, WI, USA). The clinical protocol included a non-fat-suppressed T1-weighted image and fat-suppressed T1-weighted images before and at three time-points (each 120 s apart) after the intravenous administration of 0.1 mmol/kg gadopentetate dimeglumine (Magnevist; Berlex Laboratories/Bayer Health Care Pharmaceuticals, Montville, NJ, USA) at 2 ml/s with an automatic injector (Medrad, Pittsburgh, PA, USA). Acquisition parameters were: acquisition time 120 s, repetition time 6.0 ms, echo time 4.2 ms, flip angle 10°, voxel size 0.7 × 0.7 × 3.0 mm^3^.

### Image analysis

**Fig. 1:**
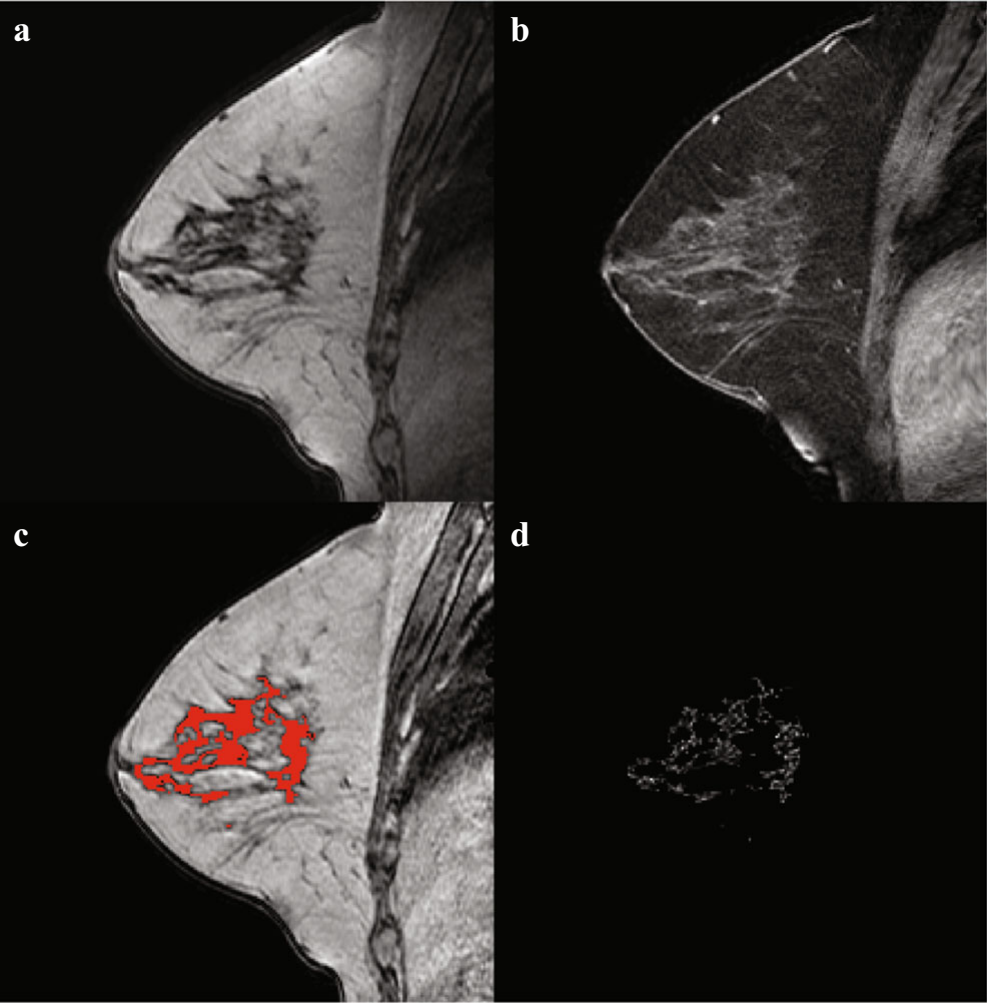
Example of the image processing in the contralateral breast of a 56-year-old patient with ER-positive/HER2-negative cancer patient. **a** non-fat-suppressed T1-weighted MRI, **b** fat-suppressed T1-weighted MRI after intravenous administration of contrast, **c** bias-field corrected image with the parenchymal tissue segmentation overlayed in *red*, **d** late enhancement in the parenchymal tissue segmentation

MRI were automatically processed using the previously published method used in the biomarker-discovery study (Fig. 1) [18]. In short, field inhomogeneities were corrected [23], the breast area was segmented [24] and the parenchymal tissue was segmented in the contralateral breast after which a morphological erosion of one in-plane voxel was used [25].

**Fig. 2:**
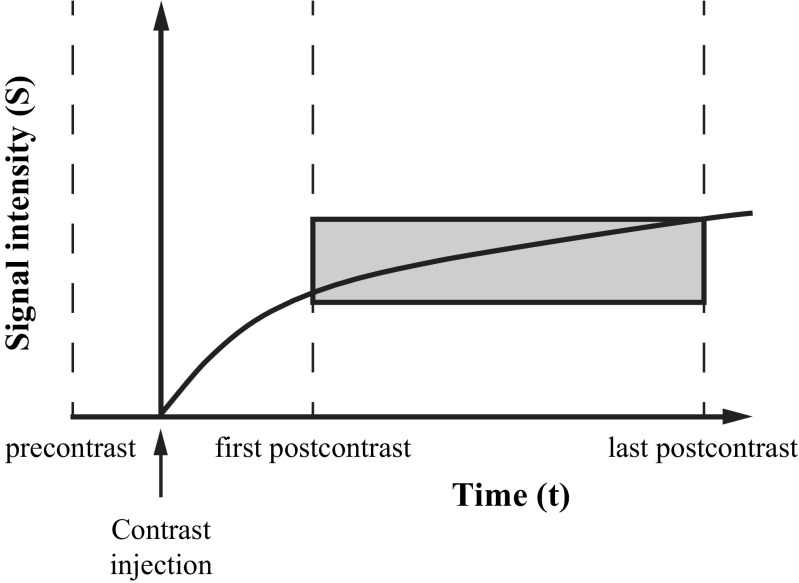
Illustration of the continuous increase in signal intensity (*vertical axis*) over time (*horizontal axis*) in the breast parenchyma after contrast injection. Late enhancement, i.e. the relative signal increase between the first and last postcontrast scan, is illustrated by the *grey box*

Dynamic contrast-enhanced series were registered to each other using deformable registration to compensate for patient motion [26]. Enhancement of the late phase was calculated at each voxel location as the relative increase in signal intensity between the first postcontrast scan and the last postcontrast scan (Fig. 2). These late enhancement values were sorted from lowest to highest, after which the top 10% was evaluated (i.e. the values above the 90th percentile). The mean of these top 10% was calculated. This top 10% parenchymal enhancement was chosen in analogy with the observation that analysis of the most enhancing part of breast lesions on MRI yields the best discriminative power between benign and malignant lesions [18, 27]. We named the quantitative unitless measure of background parenchymal enhancement of the contralateral breast *contralateral parenchymal enhancement* (CPE). This measure can be compared between patients.

### Statistical analysis

The primary endpoint of this study was to confirm that CPE reproduces as biomarker of invasive disease-free survival (IDFS) and overall survival (OS) at 10 years [28], in all ER-positive/HER2-negative breast cancer patients and in those receiving endocrine therapy. The associations were modelled using Cox proportional hazards analyses. Recurrences were coded local in case of recurrence in the ipsilateral breast parenchyma; regional in case of recurrence in the axilla, regional lymph nodes, chest wall, and ipsilateral skin; distant in all other cases [28].

In multivariable analysis, differences in patient, tumour and treatment characteristics with respect to CPE were adjusted using inverse probability weighting (IPW) based on propensity scores [29]. The characteristics adjusted for were age at diagnosis, tumour diameter, histological grade, PR status, axillary lymph node status and the type of systemic treatment administered (no therapy, endocrine therapy, chemotherapy, or endocrine and chemotherapy). Prior to IPW, CPE was trichotomised in tertiles. Thus, patients were assigned to a low, intermediate or high CPE group based on their CPE value. These groups are equal in size. In the IPW-adjusted analysis, age at diagnosis and largest tumour diameter were modelled using restricted cubic splines. Association between IPW-adjusted CPE tertiles and IDFS and OS were modelled using Cox proportional hazard analyses. Prior to the IPW-adjusted survival analyses, missing tumour characteristics underwent multiple imputations [30]. Kaplan-Meier survival curves for the IPW-adjusted CPE tertiles were generated to evaluate cumulative survival differences at 10 years; 95% confidence intervals (CIs) were assessed using 2,000 bootstrap resamples.

Baseline characteristics in patients from the biomarker-discovery study and this biomarker-assessment study were compared using unpaired *t*-tests for continuous normal variables, Mann-Whitney *U* tests for continuous non-normal variables and Fisher’s exact test for categorical variables.

To confirm the complementary value of CPE to other established biomarkers [19], we assessed the ability of CPE to find a subgroup of patients at low risk in the group of patients considered to be at high risk according to known risk models. We investigated the association between CPE and survival in patients at high risk according to the Nottingham Prognostic Index (NPI) and according to PREDICT [2, 3]. We considered patients with an NPI >3.4 at high risk, as well as patients with a 10-year overall survival below 85% according to PREDICT [2, 3].

To pursue further interpretation from potentially underlying biological processes, we explored correlations between CPE and the menstrual cycle and the percentage of hormone receptor positivity of the index-tumour in the ipsilateral breast. CPE was compared between the menopausal groups using a Mann-Whitney *U* test. CPE in premenopausal patients was compared between weeks since the start of the last menstrual cycle using Mann-Whitney *U* test. Correlations between CPE and the percentage of positive ER and PR staining on immunohistochemistry were assessed using Spearman’s non-parametric rank-test.

For all statistical tests, two-tailed *p* < 0.05 was considered significant. Analyses were performed using R 3.4.3 (R Foundation for Statistical Computing, Vienna, Austria).

## Results

### Patients

**Fig. 3:**
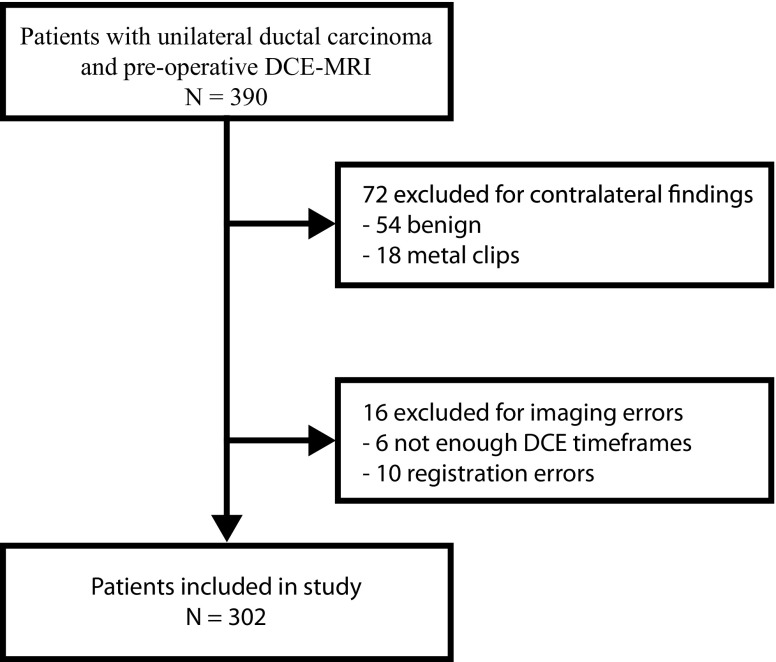
REMARK diagram flowchart of patient inclusion

Three hundred and two patients with ER-positive/HER2-negative breast cancer were included in the biomarker-assessment study (Fig. 3); 281 (93%) received endocrine therapy (Table 1). Median age at diagnosis was 48 years (interquartile range, 42-57), 10 years younger than the median age in the biomarker-discovery study. Median time of follow-up was 88 months (interquartile range, 76-102 months), comparable to the biomarker-discovery study (86 months). Fifteen of 302 (5%) patients died and 37/302 (13%) had a recurrence or died. Of these recurrences, 6/37 (16%) were local, 7/37 (19%) were regional and 24/37 (65%) were distant. The percentage of patients with IDFS (*p* = 1) or OS (*p* = 0.127) was not different from the biomarker-discovery study.

**Table 1:** Baseline characteristics in the biomarker-assessment study, compared to those in the biomarker-discovery study

Characteristic	Biomarker-assessment study (*n* = 302)	Biomarker-discovery study (*n* = 394)	*p* value^b^
Age at diagnosis (years)^a^	48 (42-57)	58 (50-64)	<0.001
Largest tumour diameter (cm)^a^	1.3 (0.8-1.9)	1.7 (1.2-2.5)	<0.001
Histological grade (%)			<0.001
Grade I	26 (9)	161 (41)	
Grade II	106 (35)	181 (46)	
Grade III	157 (52)	44 (11)	
Unknown	13 (4)	8 (2)	
Progesterone receptor			0.005
Negative	49 (16)	99 (25)	
Positive	253 (84)	294 (75)	
Unknown	0 (0)	1 (0)	
Axillary load (%)			0.001
0 positive lymph nodes	166 (55)	265 (67)	
1–3 positive lymph nodes	99 (33)	101 (26)	
4 or more positive lymph nodes	36 (12)	24 (6)	
Unknown	1 (0)	4 (1)	
Systemic therapy (%)			<0.001
No systemic therapy	12 (4)	225 (57)	
Endocrine therapy only	91 (30)	82 (21)	
Chemotherapy only	9 (3)	1 (0)	
Endocrine and chemotherapy	190 (63)	86 (22)	
Contralateral parenchymal enhancement^a^	0.37 (0.30-0.44)	0.46 (0.37-0.58)	<0.001

Values represent number of patients (percentages), unless indicated otherwise

^a^Values represent median value (interquartile range)

^b^All *p* values displayed are significant

### Multivariable survival analysis

In multivariable survival analysis, IPW-adjusted CPE was significantly associated with IDFS and OS after adjustment for age at diagnosis, histological grade, PR status, axillary load and administration of systemic therapy (Tables 2 and 3). Patients in the high CPE group had significantly better survival than patients in the low CPE group [IDFS: hazard ratio (HR) (95% CI) = 0.27 (0.05-0.68), *p* = 0.004; OS: HR (95% CI) = 0.22 (0.00-0.83), *p* = 0.032], with a cumulative IDFS at 10 years of 93% in the high CPE group compared to 72% in the low CPE group (21% difference), and a cumulative OS at 10 years of 98% compared to 82% (16% difference).

**Table 2:** Inverse probability weighting-adjusted Cox regression for invasive disease-free survival (IDFS)

	Biomarker-assessment study (*N* = 302)					Biomarker-discovery study (*N* = 394)				
		Unadjusted		IPW adjusted			Unadjusted		IPW adjusted	
All patients	*n*/*N*	HR(95%CI)	*p* value	HR (95% CI)	*p* value	*n*/*N*	HR (95% CI)	*p* value	HR (95% CI)	*p* value
		Trend-test	0.013*	Trend-test	0.004*		Trend-test	0.007*	Trend-test	0.190
Low CPE	17/101	Reference		Reference		25/132	Reference		Reference	
Intermediate CPE	15/100	0.84 (0.42-1.69)	0.63	0.84 (0.37-2.00)	0.69	15/132	0.55 (0.29-1.05)	0.070	0.66 (0.28-1.36)	0.266
High CPE	5/101	0.25 (0.09-0.68)	0.007*	0.27 (0.05-0.68)	0.004*	9/130	0.33 (0.15-0.70)	0.004*	0.59 (0.20-1.33)	0.189
Patients receiving endocrine therapy	*n*/*N*	HR (95% CI)	*p* value	HR (95% CI)	*p* value	*n*/*N*	HR (95% CI)	*p* value	HR (95% CI)	*p* value
		Trend-test	0.016*	Trend-test	0.006*		Trend-test	0.042*	Trend-test	0.022*
Low CPE	16/94	Reference		Reference		11/56	Reference		Reference	
Intermediate CPE	15/93	0.92 (0.45-1.86)	0.808	0.97 (0.42-2.16)	0.951	7/56	0.67 (0.26-1.72)	0.404	0.91 (0.22-2.62)	0.79
High CPE	5/94	0.26 (0.09-0.72)	0.009*	0.26 (0.05-0.71)	0.009*	2/56	0.18 (0.04-0.79)	0.024*	0.22 (0.00-0.79)	0.019*

Contralateral parenchymal enhancement (CPE) was split on tertiles in low, intermediate and high CPE. Differences between these CPE groups were adjusted using inverse probability weighting (IPW) for age at diagnosis, histological grade, largest tumour diameter, progesterone receptor status, axillary lymph node status and administration of endocrine and chemotherapy

In each analysis, *n* represents the number of patients that had an event and *N* the number of patients in the group analysed

Pooled hazard ratios (*HR*), 95% confidence intervals (*CI*) and *p* values are reported

Results for the biomarker-discovery study are displayed on the *right* for comparison

*p < 0.05, significant

**Table 3:** Inverse probability weighting-adjusted Cox regression for overall survival (OS)

	Biomarker-assessment study (*N* = 302)					Biomarker-discovery study (*N* = 394)				
		Unadjusted		IPW adjusted			Unadjusted		IPW adjusted	
All patients	*n*/*N*	HR (95% CI)	*p* value	HR (95% CI)	*p* value	*n*/*N*	HR (95% CI)	*p* value	HR (95% CI)	*p* value
		Trend-test	0.119	Trend-test	0.032*		Trend-test	<0.001*	Trend-test	0.080
Low CPE	8/101	Reference		Reference		21/132	Reference		Reference	
Intermediate CPE	5/100	0.59 (0.19-1.80)	0.350	0.65 (0.10-2.35)	0.44	7/132	0.29 (0.12-0.69)	0.005*	0.36 (0.09-0.86)	0.020*
High CPE	2/101	0.22 (0.05-1.06)	0.059	0.22 (0.00-0.83)	0.032*	4/130	0.17 (0.06-0.50)	0.001*	0.42 (0.05-1.10)	0.078
Patients receiving endocrine therapy	*n*/*N*	HR (95% CI)	*p* value	HR (95% CI)	*p* value	*n*/*N*	HR (95% CI)	*p* value	HR(95%CI)	*p* value
		Trend-test	0.121	Trend-test	0.026*		Trend-test	0.007*	Trend-test	0.010*
Low CPE	8/94	Reference		Reference		11/56	Reference		Reference	
Intermediate CPE	5/93	0.61 (0.20-1.87)	0.389	0.68 (0.09-2.55)	0.50	4/56	0.38 (0.12-1.18)	0.094	0.54 (0.05-1.70)	0.273
High CPE	2/94	0.22 (0.05-1.06)	0.059	0.18 (0.00-0.76)	0.024*	1/56	0.09 (0.01-0.67)	0.020*	0.14 (0.00-0.62)	0.011*

Contralateral parenchymal enhancement (CPE) was split on tertiles in low, intermediate and high CPE. Differences between these CPE groups were adjusted using inverse probability weighting (IPW) for age at diagnosis, histological grade, largest tumour diameter, progesterone receptor status, axillary lymph node status, and administration of endocrine and chemotherapy

In each analysis, *n* represents the number of patients that had an event and *N* the number of patients in the group analysed

Pooled hazard ratios (HR), 95% confidence intervals (CI) and *p* values are reported

Results for the biomarker-discovery study are displayed on the *right* for comparison

*p < 0.05, significant

**Fig. 4:**
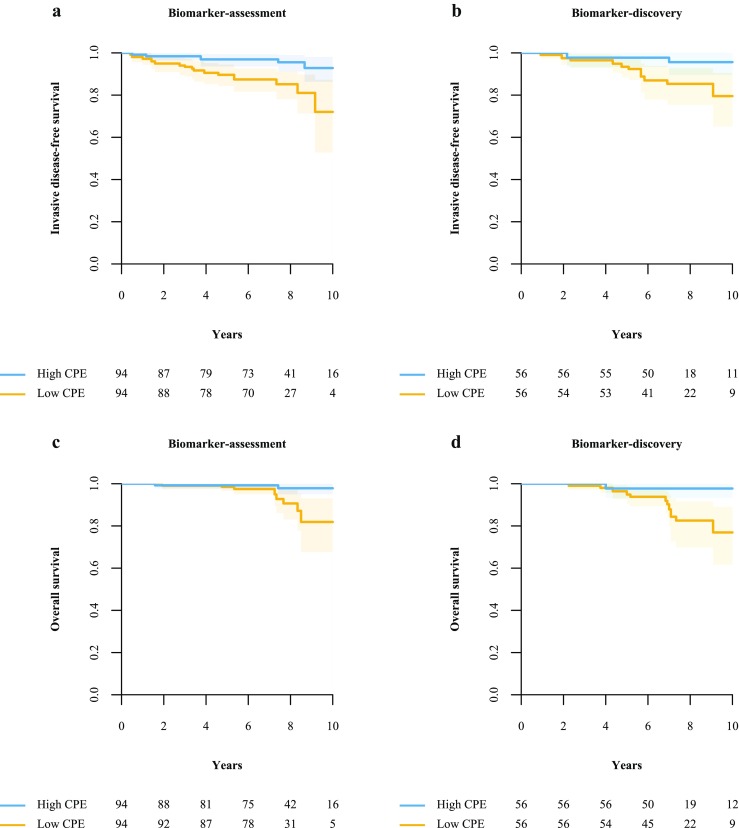
Patients with high contralateral parenchymal enhancement (CPE) have a significantly better invasive disease-free survival (IDFS) and overall survival (OS) compared to patients with low CPE in both the biomarker-assessment study and the biomarker-discovery study. Patients had an ER-positive/HER2-negative breast cancer and received endocrine therapy. At 10 years, patients in the biomarker-assessment study (**a, c**) with high CPE had a 21% higher IDFS and a 16% higher 10-year OS compared to patients with low CPE (93% and 72% for IDFS, and 98% vs 82% for OS, respectively). In the biomarker-discovery study (**b, d**), patients with high CPE had a 16% higher IDFS and a 21% higher OS compared to patients with low CPE (96% vs 80% for IDFS, and 98% vs 77% for OS, respectively). The *shaded areas* correspond to the 95% confidence intervals of the inverse probability weighting-adjusted Kaplan-Meier survival curves

For patients receiving endocrine therapy (Tables 2 and 3, Fig. 4), CPE was associated with IDFS and OS after IPW adjustment for age at diagnosis, histological grade, PR status, axillary load and administration of systemic therapy. Patients in the high CPE group had significantly better survival than patients in the low CPE group [IDFS: HR (95% CI) = 0.26 (0.05-0.71), *p* = 0.009; OS: HR (95% CI) = 0.18 (0.00-0.76), *p* = 0.024], with a cumulative IDFS at 10 years of 93% in the high CPE group compared to 72% in the low CPE group (21% difference), and a cumulative OS at 10 years of 98% compared to 82% (16% difference) (Fig. 4).

The results of this biomarker-assessment study are largely comparable with those of the biomarker-discovery study (Tables 2 and 3, Fig. 4).

### CPE with respect to established biomarkers

**Fig. 5:**
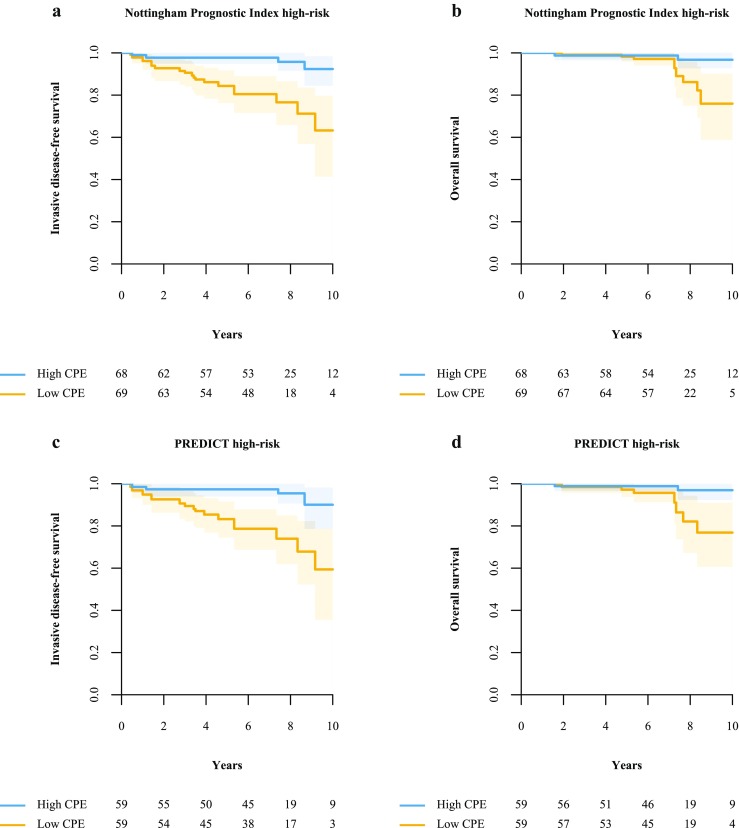
In patients considered to be at high risk according to the Nottingham Prognostic Index (**a, b**) or PREDICT (**c, d**), a subgroup with relatively good survival was identified using contralateral parenchymal enhancement (CPE); patients with high CPE (*blue curves*) had a significantly better invasive disease-free survival (IDFS) and overall survival (OS) compared to patients with low CPE (*yellow curves*). Nottingham Prognostic Index high-risk: IDFS: hazard ratio (HR) 0.15, 95% confidence interval (CI) (0.02-0.41), *p* <0 .001; OS: HR (95%CI) = 0.20 (0.00-0.83), *p* = 0.029. PREDICT high-risk: IDFS: HR (95%CI) 0.16 (0.02-0.45), *p* < 0.001; OS: HR (95%CI) 0.17 (0.00-0.76), *p* = 0.021)

In patients at high risk according to NPI (205/302, 68%), those with high CPE (*n* = 68; IDFS, four events; OS, two events) had a significantly better survival after IPW adjustment compared to the patients with low CPE (*n* = 69; IDFS: 17 events, HR (95% CI) = 0.15 (0.02-0.41), *p* < 0.001; OS: 8 events, HR (95% CI) = 0.20 (0.00-0.83), *p* = 0.029; Fig. 5).

Similar behaviour was found in patients at high risk according to PREDICT (177/302, 59%); those with high CPE (*n* = 59; IDFS, four events; OS, two events) had significantly better survival after IPW adjustment than patients with low CPE [n=59; IDFS: 15 events, HR (95% CI) = 0.16 (0.02-0.45), *p* < 0.001; OS: 7 events, HR (95% CI) = 0.17 (0.00-0.76), *p* = 0.021, Fig. 5].

### CPE and potentially related biological processes

The average value of CPE was higher in premenopausal patients than in perimenopausal and postmenopausal patients, although this difference was not statistically significant (*p* = 0.094). Out of 302 patients, 166 (55%) were premenopausal, of whom 98/166 (59%) reported their last menstrual period. CPE was not significantly associated with the timing of MRI in the menstrual cycle (*p* = 0.124). Although all patients had ER-positive breast cancer, detailed ER and PR percentages on immunohistochemistry were available in 156/302 (52%) and 153/302 (51%) patients, respectively. The correlation between CPE and ER-percentage showed a weak trend (ρ = 0.15, *p* = 0.066); CPE did not correlate with PR-percentage (ρ = 0.05, *p* = 0.51).

## Discussion

This biomarker-assessment study shows that pre-treatment contralateral parenchymal enhancement (CPE) on MRI reproduces as a biomarker of long-term patient survival. In both the previous biomarker-discovery and the current biomarker-assessment study, patients with ER-positive/HER2-negative breast cancer with high CPE in the disease-free contralateral breast had significantly better invasive disease-free survival (IDFS) and overall survival (OS) than those with low CPE after adjustment for clinicopathological parameters and systemic therapy. In addition, CPE again showed complementary ability to improve the risk stratification of routine prognostic models. This study was performed in an independent population from a different cancer centre.

In the previous biomarker-discovery study, association between CPE and patient outcome was most evident in the group of patients receiving endocrine therapy. In the current biomarker-assessment study, most patients received endocrine therapy. Hence, comparison between the groups receiving endocrine therapy is most informative. Patients who received endocrine therapy with high CPE had a fourfold to fivefold better IDFS and OS than patients with low CPE after adjustment for clinicopathological characteristics in both studies. Note that this is regardless of the lower age at diagnosis and higher tumour grade in this biomarker-assessment study. Furthermore, a different MRI vendor was used (General Electric instead of Siemens) and differences in MRI acquisition existed; the current study used fat-suppression instead of no fat-suppression and anisotropic voxels instead of isotropic voxels. Therefore, a major strength of this study is that CPE reproduces across protocol (fat-suppression vs no fat-suppression, differences in flip angle, repetition time and voxel dimensions) and vendor (General Electric vs Siemens). More recently, CPE was found to improve on the risk stratification by clinicopathological and molecular-assay based models [19]. In the current study, we confirm the potential of CPE to complement the well-established NPI and PREDICT. Confirming the complementary value of CPE to molecular assays is subject of feature research.

The biological reason why pre-treatment CPE is associated with long-term survival is not yet fully understood. CPE is a measure of the perfusion of the healthy unaffected parenchyma. We tested previously raised hypotheses concerning hormone sensitivity in this paper: we previously hypothesised that tumours in patients with high CPE have a higher hormone sensitivity and, thus, may be more receptive to endocrine therapy [18]. However, we did not find any significant correlation between ER and PR-percentage of staining at pathology and CPE, just a non-significant trend with the ER-percentage. CPE did not show a correlation with the menstrual cycle, and CPE was not significantly higher in premenopausal women than in perimenopausal and postmenopausal women. Since CPE was not significantly associated with these measures of hormone activity, they do not explain the full extent of the correlation between CPE and survival. Although further investigation into the correlation between CPE and the menopausal status may be of interest for future research, in the current study we indirectly corrected for menopausal status in our survival analysis by adjusting for age.

Other hypotheses that are subject of future research to investigate why CPE is associated with long-term survival include facilitation of drug-transport and immune response. More pronounced CPE indicates a higher parenchymal perfusion. Hence, the ability of Tamoxifen to reach loco-regional targets might be increased. A way to test this is to compare the associations of CPE with survival in patients treated with Tamoxifen and in patients treated with aromatase inhibitors, since these two anti-hormonal therapies target different mechanisms [31, 32]. High immune response is associated with superior ability of the immune system to fight residual disease [33]. Such increased immune response may be correlated with high CPE (blood perfusion in the normal parenchyma). A potential candidate to measure immune response is the cytolytic activity signature, which is associated with counter-regulatory activities limiting immune response and an improved prognosis [33]. Testing these two hypotheses is subject of future studies.

Research on the enhancement of the parenchyma includes background parenchymal enhancement (BPE), the signal enhancement ratio (SER) and texture analysis. BPE relates to the parenchymal volume and intensity enhancing on MRI and is scored in four incremental categories [34]. Increased BPE has been associated with a higher risk of breast cancer development [11, 14, 35]. SER is the quantitative signal ratio between early and late subtraction scans [36]. Increased SER has been associated with longer disease-free survival in patients after one cycle of neoadjuvant chemotherapy [12]. Although our study is not aimed at response monitoring to neoadjuvant chemotherapy, it also shows a survival benefit in patients with higher perfusion of the parenchyma. It is likely that the underlying mechanism for this observation is, however, different. Investigating associations of CPE with patient survival in a neoadjuvant setting is of interest for future research. While Hattangadi et al. [12] focused on tumour-induced changes to the parenchyma directly around the index tumour, CPE focuses on the properties of the healthy parenchyma prior to tumourigenesis. Texture analysis of parenchymal tissue has been associated with signalling pathways and patient survival [37, 38].

Our study has some limitations. Firstly, we only included patients with invasive ductal carcinoma. Therefore, we cannot generalise to invasive lobular carcinoma or other invasive types. However, the biomarker-discovery study showed no difference between CPE and survival with respect to these histological tumour types [18]. Secondly, patients with biopsy-proven benign findings in the contralateral breast were excluded. Further algorithm development is necessary to ensure that these MRI findings are not in the segmentation. Thirdly, even though this study confirms CPE as a biomarker for survival, CPE values between institutions were systematically shifted. This is likely because of differences in vendor hardware and imaging protocols, and additional precautions are required when CPE is to be trichotomised in data pooled across institutions. Fourthly, we did not always perform the MRI in the recommended menstrual window. Postponing the MRI to correct for effects of the menstrual cycle would lead to undesired delay of surgery. This limitation was, however, present in the patients alive at follow up, patients with a recurrence, and patients who had died. Therefore, we consider this potential limitation unlikely to bias the results. Lastly, these were two retrospective studies. To fully elucidate the potential of the CPE biomarker, validation in a multi-cohort prospective study is desired.

To conclude, in this retrospective cohort from an independent cancer institution, contralateral parenchymal enhancement on pre-treatment dynamic contrast-enhanced MRI reproduces as an independent biomarker of survival in patients with ER-positive/HER2-negative breast cancer. These findings are a promising next step towards a practical, widely accessible and inexpensive test for risk stratification of ER-positive/HER2-negative breast cancer that can have a practice-changing impact.

## Abbreviations

BPE: Background parenchymal enhancement
CPE: Contralateral parenchymal enhancement
ER: Oestrogen receptor
HER2: Human epidermal growth factor receptor 2
IDFS: Invasive disease-free survival
NPI: Nottingham Prognostic Index
OS: Overall survival
PR: Progesterone receptor
SER: Signal enhancement ratio
